# Higher sensitivity and accuracy of synovial next-generation sequencing in comparison to culture in diagnosing periprosthetic joint infection: a systematic review and meta-analysis

**DOI:** 10.1007/s00167-022-07196-9

**Published:** 2022-10-16

**Authors:** Ashraf T. Hantouly, Osama Alzobi, Ahmad A. Toubasi, Bashir Zikria, Mohammed Al Ateeq Al Dosari, Ghalib Ahmed

**Affiliations:** 1 Department of Orthopedic Surgery, Surgical Specialty Center, Hamad Medical Corporation, Doha, Qatar; 2 Faculty of Medicine, University of Jordan, Amman, Jordan; 3 Aspetar Orthopaedic and Sports Medicine Hospital, Doha, Qatar

**Keywords:** Periprosthetic joint infection, Arthroplasty, Diagnosis, Next-generation sequencing, Culture

## Abstract

**Purpose:**

The purpose of this meta-analysis was to compare the diagnostic parameters of synovial next-generation sequencing (NGS) and cultures in diagnosing periprosthetic joint infections (PJI).

**Methods:**

PubMed, Web of Science, Cochrane, and Google Scholar were searched from inception until 8 Jan 2022 for literature investigating the role of NGS in comparison to culture in the diagnosis of PJI. The studies were included if they investigated the diagnostic value of culture and NGS in diagnosing PJIs against the Musculoskeletal Infection Society (MSIS) criteria. Diagnostic parameters, such as sensitivity, specificity, positive predictive value, negative predictive value, positive-likelihood ratio, negative-likelihood ratio, accuracy, and area under the curve (AUC), were calculated for the included studies to evaluate the performance of NGS in comparison to culture in PJI diagnosis.

**Results:**

The total number of the included patients was 341 from seven articles. The pooled sensitivity, specificity, and diagnostic odds ratio of NGS were 94% (95% CI 91–97%), 89% (95% CI 82–95%), and 138.5 (95% CI 49.1–390.5), respectively. NGS has positive- and negative-likelihood ratios of 7.9 (95% CI 3.99–15.6) and 0.1 (95% CI 0.0–0.1), respectively. On the other hand, the pooled sensitivity, specificity, and diagnostic odds ratio of culture were 70% (95% CI 61–79%), 94% (95% CI 88–98%), and 28.0 (95% CI 12.6–62.2), respectively. The SROC curve for NGS showed that the accuracy (AUC) was 91.9%, and that the positive and negative predictive values were 8.6 (95% CI 5.0–19.5) and 0.1 (95% CI 0.0–0.1), respectively. While, culture SROC curve demonstrated that the accuracy (AUC) was 80.5% and the positive- and negative-likelihood ratio were 12.1 (95% CI 4.5–49.6) and 0.3 (95% CI 0.2–0.4).

**Conclusions:**

NGS has a potential role in diagnosing hip and knee PJIs due to its high sensitivity, specificity, and accuracy. However, the sensitivity and specificity reported by the studies varied according to the time of synovial sampling (preoperative, postoperative, or mixed).

## Introduction

Periprosthetic joint infection (PJI) is a devastating complication of joint replacement surgeries with substantial increase in mortality and morbidity [1, 11, 20]. The incidence of PJIs in primary and revision cases is 0.5–3% and 4–6%, respectively [4]. Timely and an accurate diagnosis, in addition to microorganism(s) identification, is crucial for the proper management of PJIs. However, it is still a challenge to diagnose PJIs and identify the causative organism as up to 50% of cultures fail to detect the infecting organism(s) [21]. The type of the cultured specimens has a significant impact on its reliability, as synovial fluid cultures have significantly lower sensitivity and specificity when compared to the gold standard; synovial tissue cultures [23]. Moreover, noncultivable organisms, the deleterious effects of preculture antibiotics, lack of sufficient number of organisms, and biofilm existence all play a role in the high rate of false-negative cases [2, 12, 16, 21, 22]. Culture negative PJIs (CN-PJI) lead to empiric use of antibiotics with a potential of missing the actual infecting organism [9]. Furthermore, there is a fivefold risk of reinfection with culture-negative cases when compared to culture-positive ones [14, 17]. All these limitations of using culture as a diagnostic tool for PJIs, especially when using synovial fluid instead of synovial tissue cultures, resulted in a huge inconsistency in its sensitivity, which has been reported to range between 58 and 95% and led to focus on discovering alternative methods for diagnosing PJIs [23, 27].

Synovial Next-Generation Sequencing (NGS) is an emerging technology with the ability to sequence and amplify all DNA/RNA fragments of the bacteria or even other organisms in a given sample simultaneously, giving a detailed and comprehensive picture of the microbial profile [15, 19]. This method has decreased the time needed to detect the infecting organism, and it has the potential to address the drawbacks of cultures and PJI diagnostic challenges, especially in culture-negative PJIs.

This meta-analysis aimed to evaluate the performance of NGS in diagnosing PJIs and compared it with the gold standard, cultures. The hypothesis of this study was that NGS has a higher diagnostic accuracy for PJIs when compared to cultures [5, 7].

## Materials and methods

A computer-based systematic search was performed according to the Preferred Reporting Items of Systematic Reviews and Meta-analysis (PRISMA) Guidelines [13]. PubMed, Google Scholar, Web of Science, and Cochrane databases were searched from inception until 8 Jan 2022 for literature investigating the role of NGS in the diagnosis of PJI. The following keywords were used: “Periprosthetic joint infection” OR “Prosthesis related infections” AND “NGS” OR “Next generation sequencing” OR “16S amplicon targeted sequencing” OR “metagenomic sequencing” OR “shotgun meta-genomics”. The detailed search strategy is described in supplementary material 1.

A blinded and independent process of screening based on titles and abstracts was done by two authors. Full-text review was done for the eligible studies as per the below-mentioned criteria. When discrepancies were found, a senior author gave his input to reach a consensus.

### Eligibility criteria

All articles were included if the following criteria were met:

1. Musculoskeletal Infection Society (MSIS) criteria were used to evaluate patients with suspected PJI.
2. A comparison between NGS and culture was utilized to evaluate patients with suspected PJI.
3. Sensitivity and specificity of NGS and culture were reported.

### Exclusion criteria

1. Studies that used criteria other than MSIS to identify PJI.
2. Studies that used NGS to evaluate native joints prior to a joint replacement surgery.
3. Nonaccessible articles and articles that were not published in English.

### Data collection process and data items

The following data items were collected: author’s name, study year, country of origin, age, sex, number of participants, diagnostic criteria, sensitivity, specificity, positive-likelihood ratio, negative-likelihood ratio, positive predictive value, negative predictive value, accuracy of NGS and culture, and organisms in positive NGS and culture.

### Risk of bias in individual studies

The QUADAS-2 tool was used by two independent authors to evaluate the methodological quality of the included studies. The tool is composed of four main domains; patient selection, index test, reference standard, and flow and timing [25]. The risk of bias was judged as “low”, “high”, or “unclear” based on signaling, risk of bias, and applicability rating questions. Any discrepancy in the judgment of the two authors was resolved with the input of a senior author.

### Statistical analysis

A 2 × 2 contingency table was created for all of the included studies; after that, the sensitivity, specificity, diagnostic odds ratio, positive- and negative-likelihood ratios, and positive and negative predictive values were calculated for each study. Moreover, the prevalence of the disease in the included studies was pooled using random-effect model with double arcsine transformation to use it to calculate the diagnostic parameters that need the prevalence of the disease to be calculated. The results of the mentioned diagnostic parameters of each study with their 95% confidence intervals (95% CI) were pooled using a random-effect model. Additionally, the summarized receiver-operating characteristic (SROC) curve was constructed using these diagnostic parameters. The heterogeneity of the included studies was evaluated using the Cochrane *Q* and *I*^2^ statistics. All the mentioned analysis except the SROC was conducted using Meta XL, version 5.3 (EpiGear International, Queensland, Australia). The SROC curve was generated using MetaDTA: Diagnostic Test Accuracy Meta-Analysis v2.01 [18].

## Results

### Study selection

**Fig. 1:**
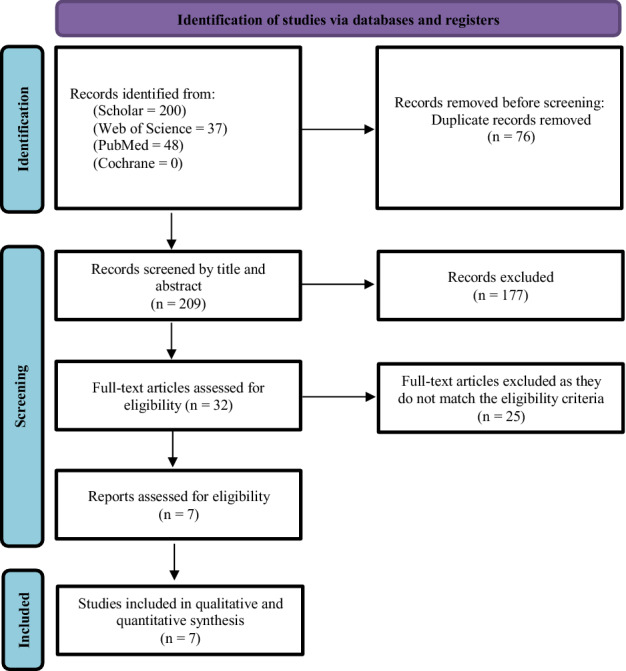
Search strategy flowchart

The search yielded 285 articles; of them, 76 were duplicates that were removed manually and electronically. The rest of the articles were screened using title/abstract and 176 of them were excluded. The remaining 33 articles were tested against the inclusion criteria using their full-text form. Finally, seven cohort articles were included in this systematic review and meta-analysis [3, 4, 6, 21, 24, 26, 28]. The detailed process of study selection is described in Fig. 1.

### Characteristics of the included studies

The total number of the included patients was 341 from seven cohort articles. The mean age and standard deviation of the patients in the included studies were 64.6 ± 12.4 and 44.28% of which were males. Among the six studies that provided data about the affected joint site, 50% of the joints were knees and the other 50% were hip joints. In addition, 58.4% of the patient’s joints were septic as per MSIS criteria, while 41.6% of them were aseptic. Moreover, three of the included studies obtained the synovial joint sample intraoperatively, and three of them used mixed preoperative and postoperative samples, whereas only one study obtained the sample preoperatively. The characteristics of the included studies are described in Table 1.

**Table 1:** Studies characteristics

Study	Country	Study design	Participants (male/female)	Age (Year ± standard deviation)	Septic (MSIS)	Aseptic (MSIS)	Sample	Sensitivity (NGS vs culture)	Specificity (NGS vs culture)	Positive predictive value (NGS vs culture)	Negative predictive value (NGS vs culture)	Positive-likelihood ratio (NGS Vs culture)	Negative-likelihood ratio (NGS Vs culture)	Accuracy (NGS Vs. culture)
He (2021) [6]	China	Prospective cohort	59 (22/37)	PJI 68.8 ± 8.1 Aseptic 63.9 ± 11.5	Knee 24 Hip 16	Knee 10 Hip 9	Intraoperative synovial fluid and tissue	95 Vs. 85%	94.7 Vs. 94.7%	94.70 Vs. 97.10%	90 Vs. 75%	–	–	–
Yin (2021) [26]	China	Prospective cohort	35 (21/14)	PJI 66.4 ± 7.6 Aseptic 68.8 ± 7.2	Knee 8 Hip 7	Knee 8 Hip 12	Preoperative synovial fluid	93.30 Vs. 46.70%	90 Vs. 95%	88 Vs. 88%	95 Vs. 70%	9.33 Vs. 9.33	0.07 Vs. 0.56	92.31 Vs. 74%
Cai (2020) [3]	China	Prospective cohort	44 (27/17)	PJI 66.4 ± 7.6 Aseptic 68.8 ± 7.2	Knee 4 Hip 18	Knee 9 Hip 13	Intraoperative synovial tissue	95.45 Vs. 72.72%	90.91 Vs. 77.27%	91.30 Vs. 76.19%	95.24 Vs. 73.91%	–	–	93.18 Vs. 75%
Fang (2020) [4]	China	Prospective cohort	38 (19/19)	PJI 63.24 ± 21.99 Aseptic 60.85 ± 14.94	Knee 6 Hip 7	Knee 13 Hip 12	Pre- and intraoperative synovial fluid and intraoperative synovial tissue	Pre-op 92% Intra-op 96% Vs. Pre-op 52% Intra-op 72%	Pre-op 92.3% Intra-op 100% Vs Pre-op 92.3% Intra-op 100%	Pre-op 95.8% Intra-op 100% Vs Pre-op 92.9% Intra-op 100%	Pre-op 85.7% Intra-op 92.9% Vs Pre-op 50% Intra-op 65%	–	–	Pre-op 92.1% Intra-op 97.4% Vs Pre-op 65.8% Intra-op 81.6%
Wang (2020) [24]	China	Prospective cohort	63	–	–	–	Preoperative synovial fluid (if insufficient, intraoperative was taken) and intraoperative synovial tissue	95.60 Vs. 77.80%	94.40 Vs. 94.40%	97.70 Vs. 97.20%	89.50 Vs. 63%	–	–	–
Zhang (2019) [28]	China	Prospective cohort	37 (20/17)	PJI 66.50 ± 8.90 Aseptic 58.36 ± 8.17	Knee 12 Hip 12	Knee 6 Hip 7	Preoperative synovial fluid (if insufficient intraoperative was taken) and intraoperative synovial tissue	100 Vs. 66.7%	100 Vs. 92.3%	96 Vs. 100%	100 Vs. 61.90%	–	–	92.31 Vs. 66.67%
Tarabich (2018) [21]	USA	Prospective cohort	65 (42/23)	PJI 63.3 ± 11.2 Aseptic 64.7 ± 10.4	Knee 13 Hip 15	Knee 26 Hip 11	Intraoperative synovial fluid and tissue	89.30 Vs. 60.70%	73 Vs. 97.30%	–	–	–	–	-

*MSIS* musculoskeletal infection society

*NGS* next-generation sequencing

### Quality assessment

**Fig. 2:**
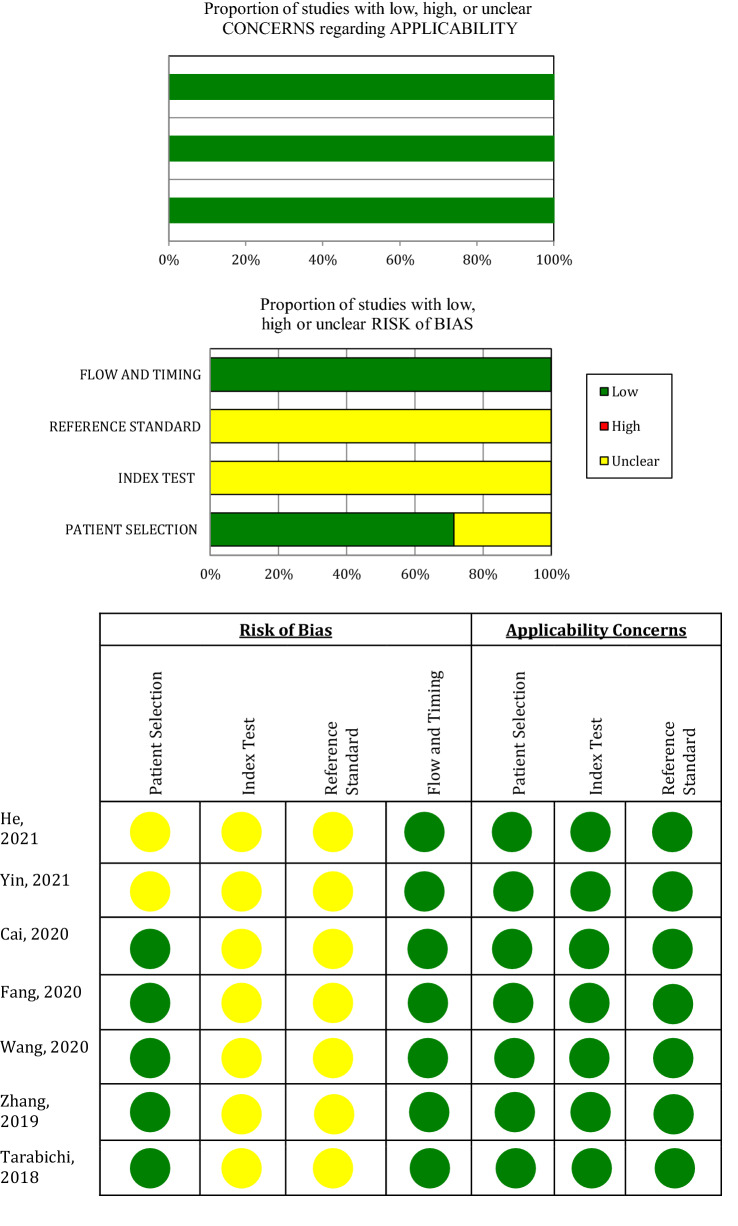
Quality assessment of the included studies using QUADAS-2 tool criteria

None of the included studies had a low risk of bias in all four domains. Two studies were unclear in the patient selection domain [6, 26]. All studies were unclear in both the index test and reference standard domains. On the other hand, all studies had a low risk of bias regarding the flow and timing domain. Low applicability concerns were found in all included studies. A summary of the qualitative assessment, according to the QUADAS-2 tool, is shown in Fig. 2.

### Next-generation sequencing and culture sensitivity

**Fig. 3:**
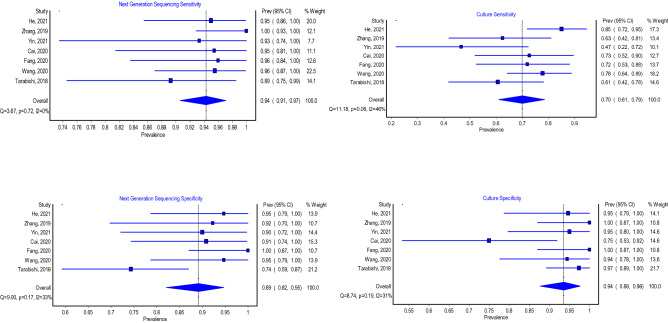
Sensitivity and specificity of next-generation sequencing and culture

Seven studies were included in the model that evaluated the sensitivity of the next-generation sequencing in diagnosing PJIs. The model showed that the overall pooled sensitivity was 94% (Fig. 3; 95% CI 91–97%); the heterogeneity of this model was not statistically significant (*P* value = not significant (NS), *I*^2^ = 0%). The highest sensitivity of the included studies was 100%, which was reported by Zhang et al. [28]. Whereas the lowest sensitivity reported was 89% and it was reported by Tarabishi et al. [21]. The model that evaluated the sensitivity of culture in diagnosing PJIs included seven studies. This model showed that the pooled sensitivity of culture was 70% (Fig. 3; 95% CI 61–79%); this model did not show a statistically significant heterogeneity (*P* value = NS, *I*^2^ = 46%). He et al. [6] reported the highest sensitivity (85%), whereas Yin et al. reported the lowest one (47%). [26].

### Next-generation sequencing and culture specificity

The model that evaluated the specificity of next-generation sequencing included seven articles. The model revealed that the pooled specificity was 89% (Fig. 3; 95% CI 82–95%) and this model showed no statistically significant heterogeneity (*P* value = 0.17, *I*^2^ = 33%). The highest specificity reported among the included studies was 100% by Fang et al. [4], while the lowest specificity was 74% by Tarabishi et al. Furthermore, the analysis showed that the overall false-positive rate of next-generation sequencing was 11% (Table 2; 95% CI 5–18.3%).

**Table 2:** Summary of next-generation sequencing (NGS) summarized receiver-operating characteristic (SROC) curve results

Parameter	Estimate	2.5% CI	97.5% CI
Sensitivity	0.940	0.906	0.974
Specificity	0.891	0.817	0.950
*θ*	1.205		
*λ*	5.797		
*β*	0.643		
*σ*_θ_	0.000		
*σ*_α_	0.791		
Diagnostic odds ratio	138.48	49.120	390.522
Positive-likelihood ratio	8.624	4.951	19.480
Negative-likelihood ratio	0.067	0.027	0.115
Likelihood ratio + ve	7.881	3.989	15.557
Likelihood ratio –ve	0.073	0.037	0.136
Accuracy	0.919		

The specificity of culture in diagnosing PJIs was also investigated by seven studies. The model that pooled these studies showed that the pooled specificity was 94% (Fig. 3; 95% CI 88–98%). This model did not show a statistically significant heterogeneity (*P* value = NS, *I*^2^ = 31%). Furthermore, the highest specificity was reported by Zhang et al. and Fang et al., as both reported a specificity of 100%. The lowest specificity was reported by Cai et al. (75%) [3]. Additionally, the analysis showed that the overall false-positive rate of culture was 5.8% (Table 3; 95% CI 1.6–13.6%).

**Table 3:** Summary of culture summarized receiver-operating characteristic (SROC) curve results

Parameter	Estimate	2.5% CI	97.5% CI
Sensitivity	0.701	0.612	0.793
Specificity	0.942	0.864	0.984
False-positive rate	0.058	0.016	0.136
Diagnostic odds ratio	28.04	12.64	62.19
Likelihood ratio +ve	8.31	3.21	21.51
Likelihood ratio –ve	0.326	0.251	0.446
Positive predictive value	12.086	4.500	49.563
Negative predictive value	0.317	0.210	0.449
Accuracy	80.5%		

### Next-generation sequencing and culture positive-likelihood ratio, negative-likelihood ratio, and diagnostic odds ratio

**Fig. 4:**
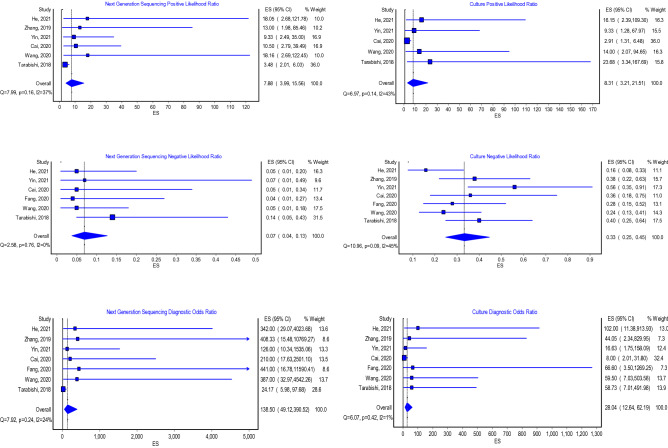
Positive-likelihood ratio, negative-likelihood ratio, and odds ratio of next-generation sequencing and culture

The model that investigated the positive-likelihood ratio (PLR) of the next-generation sequencing included six studies. The results of this model showed that the pooled PLR was 7.9 (Fig. 4; 95% CI 4.0–15.6) and the heterogeneity of this model was not statistically significant (*P* value = NS, *I*^2^ = 37%). Furthermore, six studies were pooled in the model that evaluated the negative-likelihood ratio (NLR). This model revealed that the pooled NLR was 0.1 (Fig. 4; 95% CI 0.0–0.1) and this model did not show a statistically significant heterogeneity (*P* value = NS, *I*^2^ = 0%). Additionally, the model that evaluated the diagnostic odds ratio (DOR) of the next-generation sequencing included seven studies. The model showed that the pooled DOR was 138.5 (Fig. 4; 95% CI 49.1–390.5) and this model had no statistically significant heterogeneity (*P* value = NS, *I*^2^ = 24%). The model that assessed the positive-likelihood ratio of culture in diagnosing PJIs included seven studies. This model revealed that the pooled PLR was 8.31 (Fig. 4; 95% CI 3.2–21.5); this model did not have a statistically significant heterogeneity (*P* value = NS, *I*^2^ = 43%). Moreover, the model that evaluated the negative-likelihood ratio also included seven studies. This model showed that the overall NLR was 0.3 (Fig. 4; 95% CI 0.3–0.5); this model showed no statistically significant heterogeneity (*P* value = NS, *I*^2^ = 45%). Additionally, seven studies evaluated the diagnostic odds ratio of culture in diagnosing PJIs. This model showed that the pooled DOR was 28.0 (Fig. 4; 95% CI 12.6–62.2).

### Next-generation sequencing and culture summary of receiver-operating characteristic

**Fig. 5:**
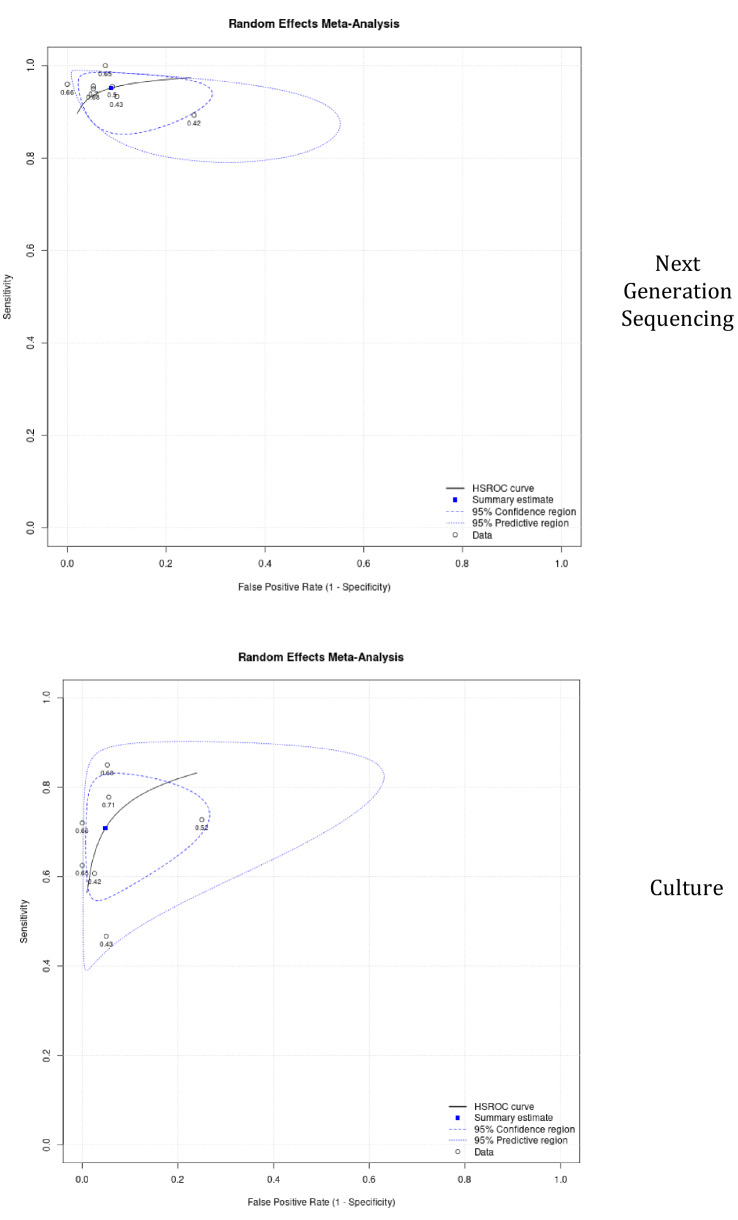
Summary of next-generation sequencing and culture receiver-operating characteristic (SROC) curves

The summary of receiver-operating characteristic (SROC) of the next-generation sequencing curve included seven studies. The SROC curve showed that the accuracy (AUC) was 91.9% (Fig. 5), and that the positive and negative predictive values were 8.6 (95% CI 5.0–19.5) and 0.067 (95% CI 0.0–0.1), respectively. Table 2 shows the summary results of the SROC curve. On the other hand, the summary of receiver-operating characteristic (SROC) of culture included seven studies. The SROC curve showed that the accuracy (AUC) was 80.5% (Fig. 5), and that the positive and negative predictive values were 12.1 (95% CI 4.5–49.6) and 0.3 (95% CI: 0.2–0.4), respectively. Table 3 shows the summary results of the SROC curve.

## Discussion

The most important finding of this meta-analysis was the excellent pooled sensitivity of NGS (94%) compared to the pooled sensitivity of culture (70%) in diagnosing of PJIs (as determined by the MSIS criteria). However, the pooled specificity of NGS (89%) is slightly lower than that of culture (94%). In addition, this study showed an excellent accuracy of NGS (91.9%) compared to a good accuracy of culture (80.5%) in the diagnosis of PJIs. Furthermore, the results of this study showed that NGS had better results in term of pooled diagnostic odds ratio compared to culture.

Comparing individual studies, the difference between the results of the included studies demonstrated that the sensitivity and specificity of NGS and culture could be affected by the timing of sampling the synovial fluid (pre- and intraoperative sampling).

In this meta-analysis, synovial fluid samples were collected preoperatively in one study [26], intraoperatively in three studies [3, 6, 21], and mixed (pre- and intraoperatively) in three studies [4, 24, 28]. Fang et al. calculated the parameters for pre- and intraoperative samples separately for both NGS and culture [4]. The sensitivity and NPV of preoperative synovial fluid for NGS (sensitivity: 92.3%, NPV: 85.7%) were significantly higher than those of preoperative synovial fluid cultures (sensitivity; 52%, NPV: 50%). However, the two groups had no significant difference in specificity or PPV. Moreover, the preoperative synovial fluid sensitivity and specificity of NGS samples were lower than those of intraoperative synovial fluid NGS (sensitivity: 92% vs. 96% and specificity; 92.3% vs. 100% for pre- and intraoperative samples, respectively). However, the differences did not reach statistical significance [4].

In the other studies, the differences in NGS sensitivity of pre- and intraoperative samples were not statistically significant, and the sensitivities were reported between 89 and 100% [3, 4, 6, 21, 24, 26, 28]. The lowest sensitivity was reported by Tarabichi et al. (89%), although tissue and synovial fluid samples were taken intraoperatively. On the other hand, sampling time significantly affected the sensitivity of culture [6, 26]. Therefore, the sample timing (pre- and postoperative) has less effect on NGS diagnostic abilities when compared to cultures.

In addition to timing of sampling (pre- and intraoperative), the type of the sampled specimen has a significant impact on the diagnostic tests’ sensitivity and specificity [23]. MSIS defined a pathogen isolated by culture from two or more separate tissue or fluid samples as one of the major criteria for diagnosing PJI. However, some studies recommend tissue sampling for culture as a gold standard for diagnosing PJIs, especially in cases of negative synovial fluid cultures with high remaining clinical suspicion [8, 10]. It is important to acknowledge that this analysis included two studies reporting the use of intraoperative synovial fluid and tissue samples [6, 21]; one study reported the use of intraoperative tissue samples [3]; three studies reported the use of both pre- and intraoperative synovial fluid and intraoperative synovial tissue samples [4, 24, 28]; and one study used preoperative synovial fluid samples solely [26]. He et al. reported the highest sensitivity (85%) for culture, using intraoperative synovial fluid and tissue [6]. The lowest sensitivity for culture was reported by Yin et al. (46.7%) where preoperative synovial fluid was used for culture [26]. However, this finding can be attributed to the fact that Yin et al. reported the use of preoperative synovial fluid (not tissue) for culture. Such findings are consistent with the literature where culture sensitivity has been reported to range between 58 and 95% [27]. Therefore, NGS results were generally more consistent and less affected by sample timing (pre- or intraoperative) or sample type (synovial fluid or tissue), which is promising in organism detection in the context of PJI.

It has been described that presampling antibiotics adversely affect culture and to a lesser extent NGS [3, 4, 6, 28]. Fang et al. reported four cases that received antibiotics prior to sampling and all of them had negative pre- and intraoperative cultures. However, NGS was positive in all of the four patients. Similarly, both Zhang et al. and He et al. reported patients with presampling antibiotics who had negative cultures but positive NGS results [5, 28]. Thus, the use of NGS in cases with presampling antibiotics can be more beneficial in detecting PJIs.

## Limitations

This study is the first systematic review and meta-analysis that investigates the role of the NGS in diagnosing PJIs. In addition, the low and not statistically significant heterogeneity across all the analysis models adds to the strength of this study. However, several limitations must be acknowledged. First, the presampling antibiotic use was not clear in most of the included studies; therefore, the NGS or culture false-negative rate might be affected. Second, the difference between the included studies in the sampling time is another limitation as some studies performed their sampling preoperatively, while other studies performed it postoperatively or in a mixed fashion and due to the low number of the included studies a subanalysis for each sampling time was not done. Third, the generalizability of our findings might be limited due to the low number of included studies and low sample size, which might result in wide confidence intervals across our outcomes. Fourth, four of the seven included studies are from the same research group with a risk that the data might contain cross-points. However, these studies had different time periods of patients’ recruitment, and some of them had different inclusion and exclusion criteria. Fifth, the low number of the included studies and low sample size limits the generalizability of our findings and resulted in wide confidence intervals across our outcomes. In addition, all the studies included infections in the knee or hip joints or both with no studies included patients with PJIs in the elbow or shoulder joints. This necessitates future prospective studies that provide details about sampling time and include patients with PJIs in the elbow or shoulder. Furthermore, the different techniques utilized in NGS might have had an impact on its diagnostic value, and hence, future studies are recommended to compare these techniques to establish the best and most reliable technique to diagnose PJIs. Finally, due to the low number of the included studies, publication bias was not assessed.

## Conclusion

Based on this meta-analysis, NGS has a potential role in diagnosing hip and knee PJIs due to its high sensitivity, specificity, accuracy, and relatively rapid turnaround time. However, the sensitivity and specificity reported by the studies varied according to the time of synovial sampling (preoperative, postoperative, or mixed).

## Data Availability

Not applicable.
